# Theoretically Based Factors Affecting Diet Quality of Preschool Children: A Cross-Sectional Study

**DOI:** 10.3390/children12020114

**Published:** 2025-01-21

**Authors:** Qutaibah Oudat, Sarah Couch, Elaine Miller, Rebecca C. Lee, Tamilyn Bakas

**Affiliations:** 1 Department of Population Health, College of Nursing, University of Cincinnati, Cincinnati, OH 45221, USA; millerel@ucmail.uc.edu (E.M.); lee2rc@ucmail.uc.edu (R.C.L.); bakastn@ucmail.uc.edu (T.B.); 2 Department of Rehabilitation, College of Allied Health Sciences, University of Cincinnati, Cincinnati, OH 45221, USA; couchsc@ucmail.uc.edu

**Keywords:** diet quality, dietary beliefs, primary caregivers, theory of planned behavior, preschoolers, feeding practices

## Abstract

Background/Objectives: Diet quality during early childhood significantly influences long-term health outcomes, including obesity and chronic disease risks. Parental feeding practices, dietary beliefs, and demographic factors have been shown to impact children’s diet quality. This study aimed to determine the extent to which the demographic characteristics and the factors of primary caregivers (dietary beliefs, intention to provide a healthy diet, feeding practices) can explain the variance in the diet quality of preschoolers in the US Methods: This descriptive correlational cross-sectional study was guided by the Theory of Planned Behavior (TPB). A total of 146 primary caregivers of preschool children (aged 3–5) were recruited through convenience and snowball sampling. Data were collected using a self-reported questionnaire and a structured telephone interview. Diet quality was assessed using the Dietary Approaches to Stop Hypertension (DASH) score. Hierarchical multiple regression analyses were conducted to determine the factors associated with preschoolers’ diet quality. Results: The mean DASH score was 40.5 (SD = 10.1), reflecting moderate to low diet quality. The results showed that 16% of the variance in diet quality was significantly explained by race (non-White) and three caregiver feeding practices (food as a reward, restriction food for health, and restriction for weight control). Of these, race (non-White) and restriction food for health were significant predictors and associated with lower diet quality in preschoolers. Conclusions: These findings align with previous studies and suggest that the conceptual framework of this study might be further refined and tested in future studies.

## 1. Introduction

Diet quality during early childhood is a fundamental determinant of long-term health, influencing physical growth, cognitive development, and the risk of chronic disease in later life [1]. The preschool years represent a critical period for establishing dietary patterns that can persist into adulthood [2,3,4], underscoring the importance of identifying factors that influence diet quality during this developmental stage.

Children’s diet quality is shaped by a combination of factors, such as demographic background, family characteristics, and parental feeding practices [5,6]. Parental feeding practices are a critical factor in shaping children’s diet quality, with certain practices either supporting or undermining healthy eating habits [7]. Positive practices, such as parental monitoring and modeling healthy eating behaviors, are associated with higher diet quality in children [8]. For example, parental monitoring has been associated with improved diet quality scores, regardless of the child’s BMI, while modeling healthy eating promotes greater consumption of fruits and vegetables [7,9,10]. Conversely, coercive practices, such as pressuring children to eat, are generally linked to poorer diet quality. Studies also indicated that children whose parents apply pressure to eat often exhibit less healthy dietary habits and lower diet quality overall [7,9,10]. In addition, demographic and household factors, such as parental BMI, education, socioeconomic status, and family size, play crucial roles in shaping children’s dietary habits [11,12]. For example, recent studies have shown children whose parents have higher education levels were more likely to have better overall diet quality [13,14]. Similarly, a study conducted in Malaysia has shown that children’s BMI and dietary intake were associated with parental BMI, indicating that parental dietary habits and body weight status significantly impact children’s diet quality [15]. Unfortunately, parental dietary beliefs and intentions to provide a healthy diet are understudied, despite their potential influence on children’s eating behaviors. While there is a growing body of evidence, limited research has theoretically investigated the unique contribution of these factors on the diet quality of preschool-aged children, particularly in the US.

Addressing these gaps is critical for designing effective interventions to promote healthier eating habits in early childhood, thereby reducing the risk of diet-related health issues, such as obesity. Guided by the Theory of Planned Behavior (TPB), this study aimed to investigate how demographic characteristics and caregiver-related factors, including dietary beliefs, intentions to provide a healthy diet, and feeding practices, influence the diet quality of preschool-aged children. By understanding these relationships, this study provides critical insights that can inform the design of culturally sensitive and theoretically grounded interventions. Additionally, our findings have implications for public health policy and clinical practice by supporting the development of targeted strategies to reduce health disparities and promote optimal feeding practices during this crucial developmental period. We hypothesized that demographic characteristics, as well as parental factors (including dietary beliefs, intentions to provide a healthy diet, and feeding practices), would significantly contribute to explaining the variance in the diet quality of preschool-aged children in the US.

## 2. Materials and Methods

### 2.1. Study Design and Conceptual Framework

**Figure 1:**
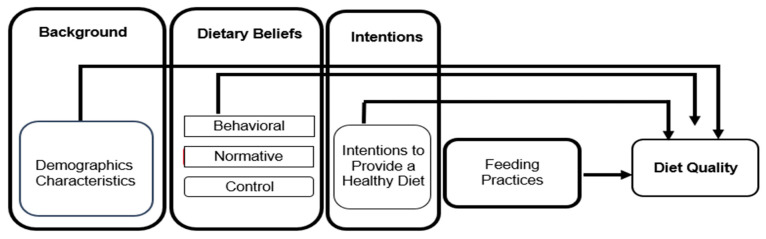
Conceptual model.

This study was a descriptive correlational cross-sectional design and was guided by a conceptual model derived from the TPB [16] (Figure 1). The TPB is a psychological theory that aims to predict and understand human behavior in specific contexts. It posits that an individual’s intention to engage in a behavior is the most immediate predictor of that behavior. This intention is influenced by several factors, including background characteristics and beliefs [16].

In our study, behavioral dietary beliefs reflected primary caregivers’ expectations, knowledge, and experiences regarding the outcomes of providing a healthy diet to preschoolers. Normative beliefs referred to the perceived social expectations, pressures, or level of support that primary caregivers received from important referents, such as family or peers, to provide their children with a healthy diet. Control beliefs captured the perceived factors that might facilitate or hinder primary caregivers’ efforts to provide a healthy diet, such as cooking skills or financial resources. Finally, the intention “intentions to provide a healthy diet” represented caregivers’ readiness and commitment to ensuring their preschoolers received a nutritious diet.

### 2.2. Study Population, Eligibility, and Sample Size Calculation

This study included primary caregivers (e.g., father, mother, grandparents) of children aged 3–5 who resided in the US. The phrase “primary caregiver” was used interchangeably with parents to refer to the person responsible for the upbringing of the preschooler, spending time with the child, supervising the child’s activities, and managing and arranging meals. Primary caregivers were eligible to participate in this study if they were at least 18 years old, able to read and converse in English, served as an unpaid primary caregiver of a child aged 3–5, were responsible for managing and arranging meals, had access to a telephone, had no hearing or talking difficulties, cared for a preschool child with no serious psychiatric or mental impairment or condition that required a special diet, had availability of time, and volunteered to participate. Pregnant caregivers were excluded from this study due to the potential impact of pregnancy on their overall diet regime, which might impact the diet of their young children.

The projected number of participants was calculated based on the formula proposed by Green (1991) (N = 50 + 8 × expected number of independent variables) [17]. The estimated sample size needed for a hierarchical multiple regression (with up to 12 independent variables) was 146 participants.

### 2.3. Sampling Techniques and Study Settings

Snowball and convenience sampling techniques [18] were used to recruit the potential study participants. Participants were recruited from three settings, including (1) daycare facilities and early childhood care and education centers; (2) community religious institutions (i.e., Churches, Synagogues, and Mosques); and (3) Facebook (www.Facebook.com). The different recruitment settings could help to recruit participants from different backgrounds to enhance the generalizability of the results across the nation [19,20].

### 2.4. Study Procedure

Flyers were posted on bulletin boards and in newsletters in the recruitment settings. Interested primary caregivers were asked to either scan the QR code embedded in the study flyer and complete the study response postcard or call the principal investigator (PI) who would complete the study response postcard on their behalf. Additionally, a Facebook group entitled “Preschool Children Diet Quality” was created; then, Facebook ads were launched from April 2022 to March 2023. Interested caregivers were asked to follow the study link attached to the Facebook ad and complete the study response postcard.

Those who completed the study response postcard were contacted to verify their eligibility, review the study information, answer any questions related to this study, and obtain their consent to be in this study. Eligible participants were asked to choose the most convenient way to receive the study questionnaire (i.e., a Redcap link sent to their email, or a mailed hard copy). Those who received the study package by mail were asked to return the study package to the PI using a postage-paid envelope when they completed the study questionnaire. The study questionnaire included questions about demographic information, dietary beliefs, intention to provide a healthy diet, and parental feeding practices. Once the participants submitted their answers and were received by the PI, they were contacted via phone to answer any missing questions and complete the Automated Self-Administered 24-h (ASA24^®^) by telephone interview. The total approximate time to complete the study questionnaire, including the single phone interview, was between 40 and 50 min.

### 2.5. Measures

Demographic Information. Demographic information was collected using a self-reported questionnaire. The caregivers were asked to report information about themselves and their children. Information was collected, such as age, sex, gender, race, ethnicity, weight (pounds), and height (inches). In this study, biological sex was defined based on physical attributes, such as chromosomes, hormones, and reproductive anatomy (e.g., male or female), while gender reflected the socially constructed roles, behaviors, expressions, and identities that individuals adopt. The reported weight (pounds) and height (inches) of the participants and their preschoolers were used to calculate their body mass index (BMI) using the imperial (English) system formula (weight (lb)/[height (in)]^2^ × 703) [21].

Dietary Beliefs and Intentions to Provide a Healthy Diet. The primary caregivers’ dietary beliefs (behavioral, normative, control) and intentions to provide a healthy diet were assessed using items developed based on the instructions of the TBP [22,23]. Items were rated on a response scale ranging from 1 to 7, with 7 being a higher endorsement of beliefs [23]. The possible score of behavioral beliefs ranged between 11 and 77, while the normative beliefs scores ranged from 4 to 28. The control beliefs facilitators ranged between 8 and 56, whereas the control hinders ranged from 4 to 28. The intentions to provide a healthy diet were rated on a scale of 1 to 7, with higher scores indicating higher intentions.

Primary Caregiver Feeding Practices. The primary caregiver feeding practices were assessed using the Comprehensive Feeding Practices Questionnaire (CFPQ) by Musher–Eizenman and Holub (2007). The CFPQ is a self-report tool with 49 items, designed to evaluate feeding practices of primary caregivers of young children between the ages of 2 and 8. The CFPQ includes 12 subscales, such as child control, modeling, and monitoring, with a Likert response scale ranging from 1 to 5 points. The total scores were computed for each subscale, and higher scores indicated greater engagement in those feeding practices [24]. Studies have shown that the CFPQ subscales have evidence of internal consistency with Cronbach’s alpha coefficients ranging between 0.52 and 0.85 [24,25,26,27].

Diet Quality (DASH Score) of Preschoolers. The Automated Self-Administered 24-h (ASA24) is a web-based tool developed by the National Cancer Institute (NCI) and used to estimate the mean intake of total energy, 65 nutrients, and 37 food groups for adults and children [28]. Additionally, it was found to be an effective tool for parents or primary caregivers to report their young children’s dietary intake [29].

The ASA24 was used in this study to gather information about the foods and drinks (including type and quantity) consumed by children between the ages of 3 and 5 years within the previous 24 h (from midnight to midnight). Primary caregivers were interviewed by telephone lasting about 15 to 25 min, while the study team entered the data into the ASA24 website. The data derived from the ASA24 were used to calculate the diet quality of preschoolers, which was measured by the Dietary Approaches to Stop Hypertension (DASH) score. The DASH score [30,31,32] assesses adherence to a dietary pattern, the DASH eating pattern, that is recommended by the 2020–2025 Dietary Guidelines for Americans as a healthy way to eat for all age groups [33].

### 2.6. Ethical Considerations

This study was approved by a university Institutional Review Board (IRB). The consent form was obtained from all recruited participants using the study information sheet (signature was not required).

### 2.7. Statistical Methods

All data were gathered and entered into Research Electronic Data Capture (Redcap), which is an encrypted web tool that is highly compliant with the Health Insurance Portability and Accountability Act (HIPAA) [34]. Gathered data were exported to IBM SPSS Statistics 28 for analyses [35]. Missing data were minimal, as participants were asked to fill in missing values during the telephone interviews for the ASA24. Missing values in the dataset were replaced with the mean score “Series mean” of the variable. Missing values exceeding 25% of any one scale or subscale were dropped to ensure outcome accuracy [36].

Descriptive statistics, including means, standard deviations (SDs), and 95% confidence intervals (CIs), summarized the data. Data quality was assessed for normality, linearity, homoscedasticity, and multicollinearity. Cronbach’s alpha was calculated to evaluate the internal consistency of dietary beliefs and feeding practices.

Guided by the conceptual model in Figure 1, a hierarchical multiple linear regression [37] was conducted to determine how demographic characteristics and primary caregiver factors (dietary beliefs, intention to provide a healthy diet, feeding practices) explained the variance in preschoolers’ diet quality (DASH score). Potential independent variables were screened using bivariate statistics (Pearson r correlation, independent samples t-test, ANOVA) and included in the model if significantly associated with diet quality. Categorical variables with more than two categories were recoded into dichotomous dummy variables. Preliminary analyses ensured that assumptions of normality, linearity, homoscedasticity, and multicollinearity were met [37].

## 3. Results

A total of 1150 individuals responded to the study advertisement. Of those, 402 (30.3%) completed the study response card and expressed a willingness to participate. Among those interested, 196 participants (48.8%) met the eligibility criteria and were sent the study questionnaire. Out of the 196 participants who received the questionnaire, 146 (74.5%) primary caregivers completed both the questionnaire and the telephone interview. The remaining 50 participants (25.5%) did not complete these steps and were, therefore, excluded from this study. Reasons for non-completion included loss of follow up and withdrawal due to a lack of incentive.

Participants had a mean age of 35.4 years (SD = 6.2), an average body mass index (BMI) of 32.2, and an average of 16.4 years of education. The majority of participants were female (95.9%), White or Caucasian (77.4%), and not Hispanic or Latino (87.7%). Most participants had only one preschool-aged child (81.5%), and 76.7% were married. Participants’ working status varied: 34.2% were employed full time, 19.9% part time, 19.2% self-employed, 8.2% unemployed, 3.4% retired, and 15.1% refused to answer. About a quarter of the participants (24.7%) reported an annual household income above USD 100,000. However, 47.3% perceived their household income as “just enough to make ends meet”, and 7.5% reported that they “do not have enough to make ends meet.” The average age of preschool-aged children was 3.9 years, with an average BMI of 17.5. The DASH score for these children ranged from 18.4 to 59.9, with a mean score of 40.5 out of a maximum of 90 (SD = 10.1), as shown in Table 1.

### 3.1. Primary Caregiver Beliefs, Intentions, and Feeding Practices

Table 2 summarizes the primary caregivers’ dietary beliefs, intentions to provide a healthy diet, and feeding practices, including mean values (M), standard deviations (SDs), ranges, and Cronbach’s alpha coefficients for reliability. Dietary beliefs were categorized into behavioral (M = 60, SD = 9.6, range 11–77, α = 0.83), normative (M = 22.3, SD = 5.5, range 4–28, α = 0.89), and control beliefs, with facilitators (M = 28.8, SD = 8, range 9–52, α = 0.71) and hindrances (M = 10.2, SD = 4.8, range 4–23, α = 0.78). Good internal consistency reliability was noted for each of the belief scales and subscales, with Cronbach alphas ranging from 0.71 to 0.89. Intentions to provide a healthy diet scored a mean of 6.4 (SD = 0.9, range 3–7). Descriptive statistics for these scales and subscales are provided in Table 2, along with intentions to provide a healthy diet that averaged 6.4 with a range from 3 to 7.

For feeding practices, Cronbach alphas and descriptive statistics were calculated for each of the 12 subscales of the Comprehensive Feeding Practices Questionnaire (CFPQ). The Cronbach’s alpha coefficients of the CFPQ subscales varied across subscales, ranging from 0.69 to 0.86 (see Table 2), except for three subscales with poor internal consistency reliability that were omitted from further analyses [encourage balance and variety (0.56); environment (0.34); and teaching about nutrition (0.31)], as shown in Table 2.

### 3.2. Relationships Between Diet Quality and Potential Independent Variables

Table 3 presents the relationship between the diet quality of preschoolers, measured by the DASH score, and various potential independent variables, including demographic characteristics and primary caregiver factors such as dietary beliefs, intention to provide a healthy diet, and feeding practices. These relationships were evaluated using bivariate statistics appropriate for each level of measurement, including Pearson correlation coefficients, independent samples t-tests, and ANOVA.

The results demonstrated a significant variation in DASH scores across racial groups, with non-White preschoolers displaying lower diet quality scores compared to their White counterparts. Additionally, three specific feeding practices by primary caregivers were significantly associated with the diet quality of preschoolers. Caregivers who restricted foods for health reasons had children with poorer diet quality [r(144) = −0.341, *p* < 0.001]. Similarly, caregivers who restricted foods for weight control also had children with poorer diet quality [r(144) = −0.212, *p* < 0.01]. Moreover, the use of food as a reward by caregivers was associated with poorer diet quality in preschoolers [r(144) = −0.214, *p* = 0.01]. Based on these findings, race and the three feeding practices—restriction for health, restriction for weight control, and using food as a reward—were identified as potential independent variables for inclusion in a hierarchical multiple regression analysis to further explore their impact on the diet quality of preschoolers.

Table 4 presents the results of a hierarchical multiple regression analysis (Enter Method) with the DASH score for preschool-aged children as the dependent variable. The potential independent variables were entered into two blocks, giving two models. In Model 1, race was the sole predictor, explaining 7% of the variability in preschoolers’ diet quality [(*p* < 0.05), adjusted R^2^ = 0.07]. This model indicated that children identified as non-White had a lower diet quality (DASH scores) (β = −0.279, *p* < 0.001). In the final model, additional variables including food as a reward, restriction of food for health, and restriction of food for weight control were included. These variables contributed an additional 10.2% to the explanation of variance in diet quality (R^2^ change = 0.102), resulting in a significant overall model that accounted for approximately 16% of the variance in diet quality (adjusted R^2^ = 0.16). Importantly, the non-White race and food restriction for health were significant predictors of the DASH score, with each contributing 4% to the unique variance in diet quality. The findings suggest that preschool-aged children of non-White caregivers and those subject to health-related food restrictions have lower diet quality scores, with standardized coefficients of −0.213 and −0.234, respectively.

## 4. Discussion

Guided by a conceptual model derived from the TPB, this study aimed to elucidate how the demographical background, family characteristics, dietary beliefs, and feeding practices of primary caregivers result in the significant variance in the diet quality of preschool children in the US. Our study is the first of its kind to carefully inspect each of the aforementioned factors, acknowledge their interconnectedness, and discuss their influence on the degree of variance in diet quality of preschool children. The findings indicated that across preschoolers of all ethnic backgrounds, the average diet quality score (DASH score) was 40.5 (SD = 10.1), suggesting an overall diet quality between moderate and low. This level of diet quality does not meet national recommendations for promoting healthy living and preventing diseases [38]. Specifically, this study found that the DASH score of preschool-aged children who identified as non-White was 21% lower (β = −0.213) compared to White participants. These findings align with previous research showing that White children generally have higher DASH scores and are more likely to have a normal body mass index (BMI) [39,40]. A recent study also demonstrated that the Healthy Eating Index (HEI) varied among races/ethnicities within every BMI category [41]. Several studies have reported a positive association between obesity incidence in non-White racial groups and poor diet quality compared to White racial groups [40,42,43]. For example, a study by de Hoog et al. (2014) found that three-year-old Black and Hispanic children consumed more sugar-sweetened beverages and fast food, indicators of poor diet quality, compared to White children [44]. Moreover, the National Health and Nutrition Examination Survey (NHANES) indicated that the prevalence of obesity in children aged 2 to 5 years was highest among Blacks (17.0%) and Hispanics (14.6%) compared to Whites (6.0%) [45]. The observed disparities in diet quality among non-White preschoolers may be partially influenced by socioeconomic factors, such as household income and caregiver education levels. For instance, lower socioeconomic status is associated with reduced access to healthy foods, limited nutrition education, and increased reliance on calorie-dense, nutrient-poor foods. Additionally, caregivers with lower education levels may have less knowledge about optimal feeding practices and dietary recommendations, further contributing to poorer diet quality in their children. While our study did not explicitly examine the interplay between socioeconomic factors and diet quality, these findings highlight the need for future research to disentangle the effects of race, income, and education on dietary behaviors. Overall, these findings along with the existing literature highlight the need for targeted interventions and policies to address racial disparities in diet quality and obesity among preschool-aged children in the US. Efforts should focus on improving access to healthy foods, promoting nutrition education, and addressing the social and environmental factors that contribute to these disparities.

The findings have also revealed that primary caregivers’ use of restricted feeding practices for health-related reasons was associated with poorer diet quality in their preschool-aged children. This aligns with previous research indicating that restrictive feeding practices, such as limiting access to certain “unhealthy” foods, can paradoxically lead to increased preference and consumption of those foods [46]. When caregivers restrict access to foods for health reasons, it may inadvertently increase children’s desire for and overconsumption of those foods, ultimately undermining the intended goal of promoting healthier eating habits. These dynamics emphasize the complex interplay between caregiver feeding practices, child food preferences, and overall diet quality. The current findings underscore the importance of supporting caregivers in adopting more positive, responsive feeding strategies that foster children’s internal regulation of hunger and satiety, rather than relying on restrictive practices. Interventions that educate caregivers on the potential unintended consequences of restrictive feeding and promote alternative approaches, such as division of responsibility in feeding [47], may be particularly beneficial for improving preschoolers’ diet quality.

Interestingly, the associations between diet quality of preschooler-aged children and other factors examined were insignificant, such as sex, income, marital status, and the primary caregiver’s dietary beliefs and intentions to provide a healthy diet. However, previous studies, especially those that included participants who received Women, Infants, and Children (WIC) benefits, indicated that household characteristics, such as household income, were associated with the diet quality of children and the variety of foods offered at home [48,49]. Other studies found that the personal characteristics of primary caregivers had a significant impact on their children’s diet quality. For example, a study stated that the body mass index (BMI) of primary caregivers was significantly associated with the diet quality and the variety of food consumption among children [50].

Although this study demonstrated a negative association between food as a reward (r = −0.34, *p* < 0.01) and poor diet quality, due to shared variance in the regression model, it was not a significant individual predictor of diet quality. Consistent with the significant moderate correlation in this study, previous studies showed a significant negative correlation between diet quality and food as a reward [51]. Additionally, other studies indicated that involvement and environmental feeding practices improved diet quality and intake of fruit in preschool-aged children [5]. Of note, the environment subscale in this study had poor internal consistency reliability as a measure and, therefore, was not analyzed further (Cronbach’s alpha = 0.34).

### 4.1. Limitations

Although the sample size of this study was appropriate for addressing its aims, several limitations should be noted. First, this study used an adapted instrument based on the TPB to evaluate caregivers’ dietary beliefs and intentions to provide a healthy diet. This adaptation may have compromised measurement validity, potentially leading to misinterpretations or limitations in the generalizability of the findings. Additionally, this study relied on self-reported data from primary caregivers, which can introduce various biases, such as recall bias. Furthermore, despite employing different methods to recruit participants, the majority of the study participants were White/Caucasian. This lack of racial diversity limits the generalizability of the findings to all racial groups. Future studies should include a more diverse and representative sample to enhance the generalizability of the results.

### 4.2. Implications for Practice

According to the study findings, the diet quality of preschool children was explained by race and restriction of food for health. Healthcare providers can support caregivers in improving the diet quality of preschool children by being culturally sensitive and aware of dietary habits influenced by different racial and ethnic backgrounds. They can provide tailored dietary advice and educate parents on the nutritional needs of preschool children, emphasizing a balanced diet rich in fruits, vegetables, whole grains, and lean proteins. Healthcare providers are encouraged to promote positive feeding practices by advising against restrictive feeding, encouraging children to make healthy food choices, and highlighting the benefits of regular family meals and a positive mealtime environment.

## 5. Conclusions

To our knowledge, our study is the first of its kind to comprehensively delve into each of the aforementioned factors and discuss their interconnected effects on diet quality variance in preschool children. Hence, we have demonstrated key insights into potential factors that might explain a significant variance in the diet quality of preschool-aged children in the US. The findings highlight the necessity for targeted interventions that address access to healthy foods and promote nutrition education, particularly among non-White populations. Additionally, the effects of restrictive feeding practices on diet quality call for a shift towards more positive and responsive feeding strategies. This study points to a complex interplay of influences on children’s diet quality, emphasizing the need for comprehensive, culturally sensitive approaches to improving dietary habits from a young age and opening the doors to further research in this understudied field.

## Figures and Tables

**Table 1:** Sociodemographic characteristics of primary caregivers and their preschoolers.

Sociodemographic Characteristics	Primary Caregivers M ± SD, Range	Preschoolers M ± SD, Range
Age	35.4 ± 6.2 (25–69)	3.9 ± 0.8 (3–5)
Body mass index (BMI)	32.2 ± 9.4 (17.3–78.8)	17.5 ± 6.6 (10.1–67.1)
Years of education	16.4 ± 4.1 (5–48)	
Diet quality (DASH score)		40.5 ± 10.1 (18.4–59.9)
	**Primary Caregivers** **n (%)**	**Preschoolers** **n (%)**
Gender		
Female	140 (95.9)	72 (49.3)
Male	6 (4.1)	73 (50.0)
Other	0 (0)	1 (0.7)
Biological Sex		
Female	140 (95.9)	73 (50.0)
Male	6 (4.1)	73 (50.0)
Number of Children Aged between 3 and 5		
1	119 (81.5)	
2	21 (14.4)	
3	3 (2.1)	
4 or more	3 (2.1)	
Smoking Status		
Never smoked	123 (84.2)	
Yes, regular smoker	10 (6.8)	
Quit or past smoker	12 (8.2)	
Other (not regular or once in a while)	1 (0.7)	
Race		
White or Caucasian	113 (77.4)	
Non-White	33 (22.6)	
Ethnicity		
Not Hispanic or Latino	128 (87.7)	
Hispanic or Latino	18 (12.3)	
Marital Status		
Never married	16 (11.0)	
Married	112 (76.7)	
Divorced	8 (5.5)	
Member of an unmarried couple	10 (6.8)	
Working Status		
Full time, 35 h or more	50 (34.2)	
Part time, less than 35 h	29 (19.9)	
Self-employed in your own business	28 (19.2)	
Unemployment compensation	12 (8.2)	
Retired	5 (3.4)	
Refuse to answer	22 (15.1)	
Total Household Income		
Less than 10,000	6 (4.1)	
10,000–19,999	7 (4.8)	
20,000–29,999	10 (6.8)	
30,000–39,999	13 (8.9)	
40,000–49,999	11 (7.5)	
50,000–59,999	15 (10.3)	
60,000–69,999	14 (9.6)	
70,000–79,000	14 (9.6)	
80,000–89,999	8 (5.5)	
90,000–99,999	12 (8.2)	
More than 100,000	36 (24.7)	
Perceived Household Income		
Comfortable	66 (45.2)	
Just have enough to make ends meet	69 (47.3)	
Do NOT have enough to make ends meet	11 (7.5)	
Perceived Concern about Current Weight		
No	64 (43.8)	
Yes	82 (56.2)	
Perceived Weight Status		
Underweight	1 (0.7)	
Normal weight	39 (26.7)	
Overweight	83 (56.8)	
Obese	21 (14.4)	
I do not know	2 (1.4)	
Following a Diet		
No	117 (80.1)	
Yes	29 (19.9)	
Relationship with Your Child		
Biological mother	129 (88.4)	
Adoptive mother	6 (4.1)	
Biological father	6 (4.1)	
Stepmother	2 (1.4)	
Grandmother	3 (2.1)	
Perceived Weight of Their Child		
Underweight		14 (9.6)
Normal weight		129 (88.4)
Overweight		2 (1.4)
Obese		1 (0.7)
Concern about their Child’s Weight		
No	133 (91.1)	
Yes	9 (6.2)	
Unsure	4 (2.7)	

**Table 2:** Primary caregivers’ dietary beliefs, intentions to provide a healthy diet, and feeding practices.

Variable	All Primary Caregivers (N = 146) M ± SD	Range	Cronbach’s Alpha
Dietary beliefs			
Behavioral	60 ± 9.6	11–77	0.83
Normative	22.3 ± 5.5	4–28	0.89
Control			0.71
Facilitators	28.8 ± 8	9–52	0.78
Hindrances	10.2 ± 4.8	4–23	0.71
Intentions To Provide a Healthy Diet	6.4± 0.9	3–7	
Feeding Practices			
Control	15.4 ± 3.5	6–25	0.69
Emotion regulation	5.6 ± 1.9	3–11	0.69
Encourage balance and variety	18.2 ± 1.8	11–20	0.56
Environment	13.3 ± 1.8	10–18	0.34
Food as a reward	7.6 ± 3.2	3–14	0.73
Involvement	11.7 ± 2.7	4–15	0.69
Modeling	17.2 ± 3.3	4–20	0.86
Monitoring	14.5 ± 4.1	4–20	0.87
Pressure	9.9 ± 3.8	4–19	0.77
Restrictions for health	13.3 ± 4.8	4–20	0.86
Restriction for weight control	12.2 ± 5.6	8–40	0.87
Teaching about nutrition	10.1 ± 2.0	3–15	0.31

**Table 3:** Analyses of preschoolers’ diet quality (DASH scores) using Pearson correlation, independent samples t-tests, and ANOVA across demographic and caregiver factors.

Variable (s)	Test	Sig
	Pearson Correlation (r)	
Demographics		
Age		
Parent	0.096	0.248
Children	−0.100	0.232
BMI		
Parent	−0.156	0.060
Children	−0.137	0.100
Years of education (years)	0.144	0.085
	Independent Sample Test t-value	
Gender		
Parent	1.280	0.202
Child	0.885	0.378
Sex		
Parent	1.280	0.202
Child	0.911	0.364
Concern about current weight	0.476	0.635
Following a diet	1.633	0.105
	ANOVA (F-value)	
Smoking status	0.732	0.530
Race	2.865 *	0.017
Ethnicity	0.900	0.344
Marital status	2.013	0.115
Working status	0.344	0.885
Household income	0.861	0.571
Considering household income	0.702	0.497
Consider your current weight	1.005	0.407
Number of children between 3 and 5	0.361	0.782
Relationship to the child	0.519	0.722
Concern about child’s weight	0.311	0.733
Perceived child’s weight	1.670	0.176
	Pearson Correlation (r)	
Dietary Beliefs		
Behavioral	0.007	0.933
Normative	0.099	0.233
Control		
Facilitators	−0.089	0.285
Hindrances	−0.140	0.910
Intentions to Provide a Healthy Diet	0.152	0.068
Feeding Practices		
Child control	−0.139	0.095
Emotion regulation	−0.070	0.399
Food as a reward	−0.214 **	0.010
Involvement	0.029	0.731
Modeling	−0.030	0.720
Monitoring	−0.070	0.402
Pressure	−0.119	0.153
Restriction for health	−0.341 ***	<0.001
Restriction for weight control	−0.212 **	<0.01

* *p* < 0.05. ** *p* < 0.01. *** *p* < 0.001.

**Table 4:** Hierarchical regression with the diet quality (DASH score) of preschoolers.

Independent Variables	Standardized Beta	*p*-Value	Bivariate r	Unique r^2^
Demographic Variables				
Non-White races	−0.279	<0.001	−0.279 ***	0.08
Model 1. R= 0.28; adjusted R^2^ = 0.07; R^2^ changed= 0.08, *p* < 0.05				
Demographic Variables				
Non-White races	−0.213	0.007	−0.279 ***	0.04
Feeding Practices				
Restriction for weight control	−0.088	0.282	−0.212 **	0.01
Restriction of food for health	−0.234	0.008	−0.341 ***	0.04
Food as a reward	−0.106	0.201	−0.214 **	0.01
Model 2. R = 0.42; adjusted R^2^ = 0.16; R^2^ changed = 0.102, *p* < 0.05				

* *p* < 0.05. ** *p* < 0.01. *** *p* < 0.001.

## Data Availability

The data supporting the reported results of this study are not publicly available due to ethical restrictions and the protection of participant confidentiality. Access to the data may be considered on a case-by-case basis and can be obtained by contacting the corresponding author.
